# TPMS: a set of utilities for querying collections of gene trees

**DOI:** 10.1186/1471-2105-14-109

**Published:** 2013-03-27

**Authors:** Thomas Bigot, Vincent Daubin, Florent Lassalle, Guy Perrière

**Affiliations:** 1 Laboratoire de Biométrie et Biologie Évolutive, UMR CNRS 5558, Université Claude Bernard – Lyon 1, 43 bd. du 11 Novembre 1918, 69622 Villeurbanne Cedex, France

## Abstract

**Background:**

The information in large collections of phylogenetic trees is useful for many comparative genomic studies. Therefore, there is a need for flexible tools that allow exploration of such collections in order to retrieve relevant data as quickly as possible.

**Results:**

In this paper, we present TPMS (Tree Pattern-Matching Suite), a set of programs for handling and retrieving gene trees according to different criteria. The programs from the suite include utilities for tree collection building, specific tree-pattern search strategies and tree rooting. Use of TPMS is illustrated through three examples: systematic search for incongruencies in a large tree collection, a short study on the Coelomata/Ecdysozoa controversy and an evaluation of the level of support for a recently published Mammal phylogeny.

**Conclusion:**

TPMS is a powerful suite allowing to quickly retrieve sets of trees matching complex patterns in large collection or to root trees using more rigorous approaches than the classical midpoint method. As it is made of a set of command-line programs, it can be easily integrated in any sequence analysis pipeline for an automated use.

## Background

Comparative genomics is a central approach in sequence analysis, and many important biological results have been obtained through its use. Among the different programs and packages developed for comparative genomics, those using the information contained in phylogenetic trees are of special interest. Indeed, the explicative power brought by trees has still no match for problems like orthology detection, Horizontal Gene Transfer (HGT) prediction, or large-scale evolutionary studies. For orthologs detection, the most rigorous approach to determine whether homologous genes are orthologous or paralogous consists in comparing a gene tree to the species tree considered as a reference [1-4]. Similarly, the same approach can be used for HGT detection [5]. Lastly, various kind of evolutionary studies have been made possible only through the use of large collection of trees, *e.g.*, detection of positive selection in vertebrate evolution [6], analysis of DNA methylation levels in mammals [7], or GC-content evolution [8].

The problem is that manual browsing of collections containing thousands of gene trees is a tedious – and now, almost impossible to perform – task, and automated tools are required for large scale studies. Therefore, we have developed TPMS, a set of programs devoted to tree manipulation and query. Its central functionality is a pattern-matching algorithm aimed at finding gene trees containing specific motifs. Those patterns are written using an extended Newick format and they usually include some kind of constraint, such as node nature (duplication, speciation), subtree content, or level of statistical support for the nodes (*e.g.*, bootstrap, jackknife or aLRT). The second main functionality brought by TPMS is a tree-rooting tool based on the use of a gene unicity score and taxonomic criteria. Its aim is to place the root of a gene tree in order to minimize the incongruencies observed between this tree and a reference species tree.

The tree pattern search algorithm used by TPMS is an improved version of the one we previously published [1]. The main improvement is the possibility to query tree collections built by the users. Our previous version was implemented in FamFetch, a Graphical User Interface (GUI) that was only able to query a set of predefined databases installed on a centralized server [9].

## Implementation

TPMS is a set of C++ command-line programs that require the Bio++ [10] and Boost (http://www.boost.org/) libraries to run and it is not dependent on the use of the gene families databases developed in our group [9]. Binaries are provided for Linux and MacOSX (Intel architectures only), as well as C++ source code and documentation.

### Tree collection building

TPMS needs to be run on a collection built in the RAP format [1]. With this format, all the trees are included in a single text file. The header of this file consists in a reference species tree in Newick format that contains taxa names on its internal nodes and leaves. Names on leaves correspond to species and names on internal nodes correspond to higher taxonomic groups. All taxonomic groups allow the use of synonyms. For instance, in the example file distributed with TPMS, the leaf corresponding to *Escherichia coli* strain K12 substrain W3110 is written as:

The different synonyms are separated by a slash and it is possible to use any of them when building a query.

Individual gene trees are listed after the reference species tree. Each entry is written in Newick format and has associated information consisting in the tree name and a list of associations between the sequences names used at the leaves and their corresponding species names:

The species tree can contain unresolved nodes (multifurcations), but not the individual gene trees since the tree pattern matching algorithm used only supports binary trees [1].

The program *tpms_mkdb* is provided in order to produce a collection in the suitable format. To run this program, the minimal requirements are a species tree file and a collection of gene trees in Newick format. Using the information provided in these two files, the user must then build another file containing the association between all the sequences names used in the gene trees and the species to which they belong. Note that *tpms_mkdb* provides a functionality for facilitating this task. This functionality returns a ready-to-complete list of all the sequences names found in the gene trees files. Then the user has to fill the blanks with corresponding species names.

### Tree pattern-matching

The tree pattern-matching algorithm itself is implemented in the *tpms_query* program. This is a C++ version of the one used in FamFetch, and a complete description of the algorithm can be found in [1]. Like in the original program, both the target tree and the tree pattern need to be rooted.

Tree patterns have to be written in an extended Newick format, and they can include different criteria. Let *υ* be a node from the pattern *P*, and let *ω* and *φ* be the two sons of *υ*. In our format, *υ* is described by three strings separated by slashes. The first two strings contains the constraints put on *υ*, whereas the third one – which is only suitable for leaves – describes the set of taxa allowed at this level. The simplest pattern only contains the list of taxa to be found on the leaves with their respective positions. For instance, the pattern ((//Homo sapiens,//Pan troglodytes),//Rodentia) will find all the gene trees in which a subtree with sequences from *Homo sapiens* and *Pan troglodytes* species are grouped, while sequences from Rodents are located outside of this group. It is possible to accurately ask for the inclusion or the exclusion of specific taxa through the use of + and − signs. In this case, the pattern ((//Homo sapiens,//Pan troglodytes),//Mammalia −Primates) will find all the gene trees in which a subtree with sequences from *H. sapiens* and *P. troglodytes* species are grouped, while any sequences from Mammals that are not from Primates are located outside this group. This pattern is less constrained than the first one, and the trees it selects will include all those selected by the first one.

**Figure 1:**
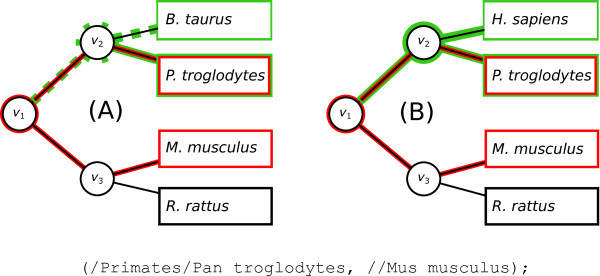
**Example of a subtree constraint.** In the pattern shown in the lower part of the figure, a constraint has been put on the node leading to *P. troglodytes*. The pattern is shown in red, and the subtree constraint is shown in green. This pattern with this constraint will find all the trees in which a subtree with sequences from *P. troglodytes* and *M. musculus* species are grouped. Non-Primates are forbidden from *ν*_1_, the common ancestor of *P. troglodytes* and *M. musculus*, to the node matching to *P. troglodytes*. Tree (**A**) is rejected, since the pattern does not allow *B. taurus* (which is not a Primate) to be in the subtree containing *P. troglodytes* (generated by *ν*_1_). On the other hand, tree (**B**) is accepted, as the subtree generated by *ν*_1_ contains only primates.

It is also possible to introduce taxonomic constraints on one of the two sons of the node matching to *υ* in the gene tree. This constraint is written on *ω*, but is put on its father node since the node matching on *ω* is not necessarily the direct son of the node matching on *υ*. This kind of constraint is put after the first slash, and examples of accepted and rejected topologies for a simple pattern are given in Figure 1.

Constraints other than purely taxonomic ones can be introduced on nodes. First, it is possible to search for patterns including a threshold for internal branches statistical support. For instance, the pattern (//Homo sapiens, //Mus musculus)$90 will select all the gene trees in which a subtree with sequences from *H. sapiens* and *Mus Musculus* are grouped, this with an associated bootstrap ≥90*%*. Also, constraints on speciation or duplication can be set on nodes if the program is running on a reconciled trees collection. This kind of nodes can be specified by the use of letters S or D. The pattern (//Homo sapiens, //Mus musculus)D will find all the gene trees in which a subtree with sequences from *H. sapiens* and *M. musculus* are grouped, while the node that groups them is a duplication node. Lastly, it is possible to specify that a connection between two nodes in the gene tree is direct. This is specified through the use of the exclamation mark. Therefore, the pattern (!//Homo sapiens, //Mus musculus) will retrieve only gene trees in which the common ancestor of *H. sapiens* and *Mus musculus* has the leaf *H. sapiens* as a direct son.

### Tree rooting

Tree rooting is an essential step in phylogeny as it orients the tree and enables the evolutionary history it summarizes to be interpreted. We have therefore implemented two rooting strategies in the *tpms_computation* program: the first one aims at maximizing the size of subtrees with unicopy sequences while the second one tries to maximize the accuracy of taxonomic affectations for nodes. The first approach introduces a score on the internal nodes of a rooted tree. Given a node $\nu_{i}\;(i=1,2,\ldots ,n-1)$, its score *u*_*i*_ is obtained by the product of the number of sequences by species encountered in the subtree generated by the node considered. A score equal to 1 means that the corresponding subtree only contains one sequence for each species. Now, let *T* be a rooted tree, its unicity score *U*^(*T*)^ is equal to:

(1) $$U^{\left(T\right)}=\overset{n-1}{\underset{i=1}{\sum }}\ln\left(\overset{\left(T\right)}{\underset{i}{u}}\right)$$

**Figure 2:**
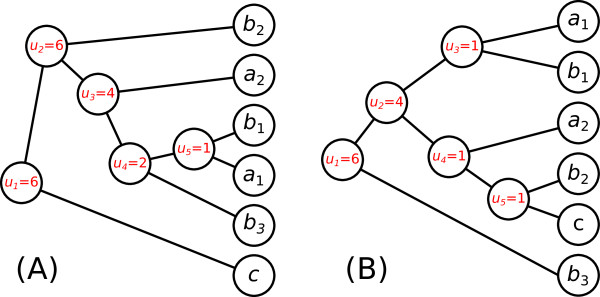
**Example of unicity score computing for a gene tree with two different rootings.** In this tree, we have six sequences *a*_1_, *a*_2_, *b*_1_, *b*_2_, *b*_3_ and *c*. Sequences *a*_1_ and *a*_2_ belong to species *A*; *b*_1_, *b*_2_ and *b*_3_ belong to species *B*; and *c* belongs to species *C*. Scores of internal nodes are given in the circles. In this example, the rooting for topology (**A**) gives the unicity score *U*^(*T*)^= ln(6)+ ln(6)+ ln(4)+ ln(2)+ ln(1)≈5.663, and the rooting for topology (**B**) gives the score *U*^(*T*)^= ln(6)+ ln(4)+ ln(1)+ ln(1)+ ln(1)≈3.178. The rooting in (**B**) is therefore preferred over the one in (**A**).

In a unrooted tree with *n* leaves, there are 2*n*−3 possible positions for the root. TPMS will try every position, and for each one, computes the unicity score of the corresponding rooted tree. Then, the rooting that is kept is the one that minimizes *U*^(*T*)^. This approach favors duplications closer to the root than to the leaves. It minimizes the number of duplication events, and is therefore based on a parsimony reasoning. This method can be useful to extract unicopy subtrees from gene families containing many copies for some species. It can automatically split trees containing paralogs into several subtrees. Figure 2 shows two rooting examples of a simple gene tree containing six sequences from three different species.

The second rooting method uses the reference species tree included in the header of the tree collection. During the rooting procedure, each internal node *ν*_*i*_ of the gene tree is associated with the taxonomic group corresponding to the Lowest Common Ancestor (LCA) in the species tree. Then, a distance *d*_*i*_ is computed for each node. This distance is equal to the number of nodes between the root of the species tree, and the node of the taxonomic group in the species tree associated with the node in the gene tree. Note that the taxa from the species tree that are not present in the gene tree are not taken into account in this distance computation. Therefore, the sum of node distances for a rooted tree *T* is equal to:

(2) $$D^{\left(T\right)}=\overset{n-1}{\underset{i=1}{\sum }}d_{i}^{\left(T\right)}$$

**Figure 3:**
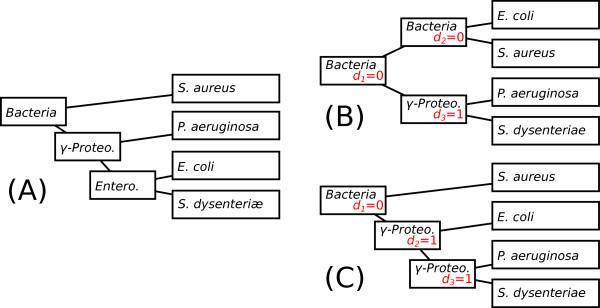
**Example of distance score computing for a gene tree with two different rootings.** The reference species tree is shown in (**A**), with the names of the taxonomic groups written on the nodes (Bacteria, *γ*-Proteobacteria and Enterobacteria). The rooting given in (**B**) gives a sum of distances *D*^(*T*)^=1+0+0=1, while the rooting given in (**C**) gives a sum of distances *D*^(*T*)^=1+1+0=2. The rooting in (**C**) is therefore preferred over the one in (**B**).

Again, TPMS will try all the 2*n*−3 rooting positions and will keep the one maximizing *D*^(*T*)^. Indeed, higher *D*^(*T*)^ values correspond to better taxonomic affectations (*i.e.*, resulting trees that are more congruent to the reference species tree). Figure 3 shows an example of two possible rootings for a bacterial gene tree, this considering a reference species tree.

Usually, the two approaches are used in sequence, the first one is efficient to isolate clusters of unique sequences, but it can lead to many *ex aequo*. The problem is that, among those *ex aequo*, some of them can contain obviously erroneous polyphyletic groups. On the other hand, the second approach will find the solutions that are more consistent to the species tree. It can be therefore used to solve those *ex aequo* in the right way, as it will remove the solutions that are in violation of the reference. If more candidates still remains after those two steps, the root is placed on the solution having the longest branch. In case of branch lengths equality, placement is arbitrary on one of the remaining possibilities.

## Data sets

For the detection of tree incongruencies in complete genomes, we performed the search on the 128674 gene trees available from HOGENOM release 5 (http://pbil.univ-lyon1.fr/databases/hogenom/). Those trees include a total of respectively 2789275 bacterial, 138474 archaeal and 738819 eukaryotic sequences. For the Ecdysozoa/Coelomata controversy and the mammals phylogeny study, we used the 14190 gene trees available from HOMOLENS release 5 (http://pbil.univ-lyon1.fr/databases/homolens.php). For the first two studies, the reference species tree used was the one provided by NCBI (http://ftp.ncbi.nih.gov/pub/taxonomy/) and for the mammal phylogeny we used a slightly modified version of the tree published by Dos Reis *et al.*[11]. Those modifications were made to match the species tree available in HOMOLENS as three species from [11] were not available in this database: *Pongo abelii*, *Vicugna pacos* and *Canis familiaris*. They were replaced respectively by *Pongo pygmaeus*, *Lama pacos* and a clade grouping *Canis lupus familiaris* and *Ailuropoda melanoleuca*.

## Results

### Incongruencies detection

The program *tpms_computation* can be used to detect taxonomic incongruencies. As seen above, each node in a gene tree can be assigned to its LCA, this using the information provided by the reference species tree. To detect the incongruencies, the approach used is to see if a leaf or a node in a gene tree induces a perturbation in this taxonomy affectation. Exploration of the tree starts from the leaves and goes up to the root. For each node, the algorithm masks the subtree it generates. If this masking leads to a taxonomic assignation change on its grandfather node, then this subtree is pruned. A perturbation index is associated to this incongruency, it correspond to the distance (in nodes numbers and with ignoring the taxa that are not present in the gene tree) between the former taxonomic affectation and the one realized after the masking process. This operation is repeated until all the existing incongruencies have been detected. Lastly, to be considered as a true incongruency, the involved branch must have a support value greater than a threshold given by the user.

**Figure 4:**
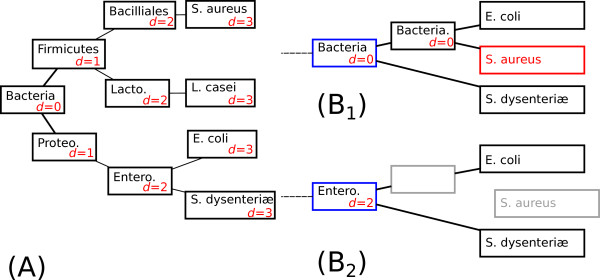
**Example of incongruency detection in a subtree.** The reference species tree is shown in (**A**), with the names of the taxonomic groups written on the nodes. For each internal node, the distance *d* to the root is written in red. The subtree containing the incongruency is shown in (**B1**), with *S. aureus* being the misplaced taxon. In this subtree, the taxonomic group associated to the grandfather of *S. aureus* is Bacteria and its distance to the root in the species tree is *d*=0. In (**B2**) the father node of *S. aureus* is removed. The root of this subtree has now two sons *E. coli* and *S. dysenteriae*. The new taxonomic affectation of this node becomes Enterobacteria, which corresponds to a distance to the root *d*=2, in the species tree. Therefore, perturbation induced by the misplacement of *S. aureus* is equal to 2−0=2.

A simple example of detection is given in Figure 4. In this example, *Staphylococcus aureus*, a Firmicute, is wrongly grouped with *Escherichia coli*, an Enterobacteria. Masking the node leading to *S. aureus* leads to a change from Bacteria to Enterobacteria in the taxonomic assignation of its grandfather node. As the number of nodes separating Bacteria from Enterobacteria in the species tree used, the perturbation index associated with this incongruency is equal to 2.

Over the 128674 gene trees available in HOGENOM, *tpms_computation* found 110359 incongruencies supported by bootstrap values ≥90*%*, this corresponds to an average of 0.86 errors per tree. Different events could explain the incongruencies observed: phylogenetic reconstruction errors (such as long branch attraction artefacts), errors in the NCBI species tree, wrong gene tree rooting by TPMS, hidden paralogies (as the gene trees from HOGENOM are not reconciled), incomplete lineage sorting and HGTs. Due to that fact, it is remarkable that their frequency is relatively low, with less than 1 error per tree on average.

**Figure 5:**
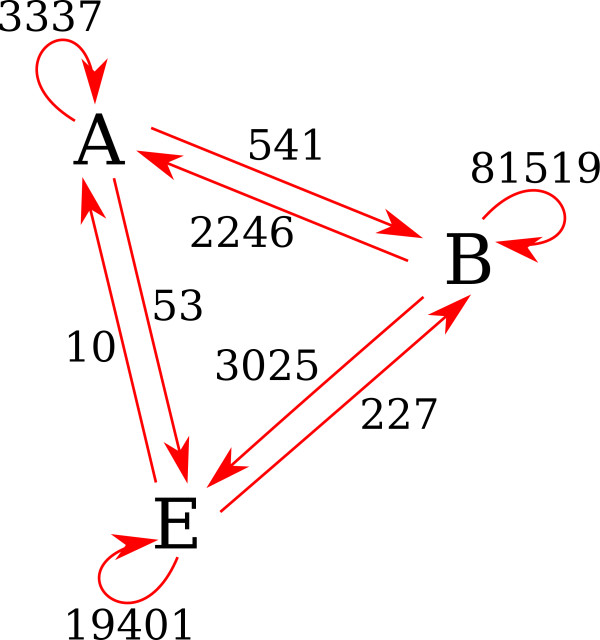
**Number of incongruencies inferred between and inside the three domains of life.** The three domains (Archaea, Bacteria and Eukaryota) are represented respectively by the letters A, B and E. For inter-domain incongruencies, the arrows give the direction of the switch observed.

The number of intra and inter-domain incongruencies detected is shown in Figure 5. An intra-domain incongruency is when a species is placed inside its domain but outside the taxonomic group it belongs to and an inter-domain incongruency is when a species is wrongly located in another domain than the one it belongs to. A good way to see if our method can be used to detect HGTs is to look at its ability to identify well-known transfers such as the ones observed between mitochondria or chloroplast and the nucleus of eukaryotes. For that purpose, we looked at the incongruences found with the 374 eukaryotic genes labelled as mitochondrial in HOGENOM 5. Among those genes, 252 (67.4%) were found to be involved in an incongruency placing them among Bacteria. Similarly, among the 377 genes labelled as chloroplastic, 249 (66%) were found to be involved in an incongruency placing them among Bacteria.

### Coelomata/Ecdysozoa controversy

The issue of whether coelomates form a single clade, the Coelomata, or whether all animals that moult an exoskeleton (such as the coelomate arthropods and the pseudocoelomate nematodes) form a distinct clade, the Ecdysozoa, is still a major open-ended subject in evolutionary biology. While single-gene based phylogenies supported the Ecdysozoa hypothesis [12-14], a first wave of phylogenomic analyses mostly favored the Coelomata [15-17]. More recently, those results were challenged by other genomic-scale studies [18-20], shifting again the balance toward Ecdysozoa. Here we present a short example showing how it is possible to retrieve the trees supporting either hypothesis.

Using TPMS, the patterns to find are (//Fungi,(/Nematoda/Nematoda,(/Chordata/Chordata, /Arthropoda/Arthropoda)$80)$80)and(//Fungi,(/Chordata/Chordata,(/Nematoda/Nematoda,/Arthropoda/Arthropoda)$80)$80), respectively for Coelomata and Ecdysozoa hypotheses with a bootstrap support ≥80*%*. We performed the search into a collection built with the HOMOLENS database, this with several thresholds for bootstrap values (Table 1). Excepted for the search performed with no minimal value for bootstrap scores, the results returned were systematically in favor of the Coelomata hypothesis.

**Table 1:** Number of tree patterns matching Coelomata or Ecdysozoa hypotheses

	**no thr**.	***≥*80*%***	***≥*85*%***	***≥*90*%***	***≥*95*%***
Coelomata	768	241	174	110	54
Ecdysozoa	1309	149	83	45	20

The queries corresponding to the two hypotheses have returned the numbers of patterns shown in the table. Five different thresholds have been used for bootstrap, from no threshold to values higher than 95%.

Note that this small example is only aimed at showing the possibilities provided by TPMS in terms of tree pattern search. To fully validate the result presented above it would be necessary to go back to the sequence alignments and test for the relative evolutionary rates of individual genes, this in order to avoid systematic biases leading to reconstruction artefacts such as long branch attraction. Indeed, the only nematode genome available in HOGENOM 5 is *Caenorhabditis elegans*, and this organism is known to have genes with high evolutionary rates, leading frequently to such kind of artefacts [21].

### Mammals phylogeny

In order to find sets of orthologs in a specific taxon it is possible to query a collection using a pattern corresponding to the subtree of a species tree containing the given taxon. If a gene tree contains exactly this pattern, then the most parsimonious hypothesis is that the matching sequences are orthologous. The search for exact patterns in TPMS requires a direct link between nodes, which is specified by the exclamation mark. For instance, to query a collection to find orthologous genes inside the *Rodentia* order, we must select all the gene trees containing the TPMS pattern ((((!//Rattus Norvegicus,!//MusMusculus)!, !//Dipodomys ordii)!, !// Cavia Porcellus) !, !// SpermophilusTridecemlineatus). This feature can be used to test the support level for nodes in a species tree. As an example, we used the mammals phylogeny published by Dos Reis *et al.*[11]. For that purpose, we extracted all possible subtrees in this tree and used the corresponding patterns to perform searches in HOMOLENS. Complete listing of the patterns in TPMS format is provided as Additional file 1.

**Figure 6:**
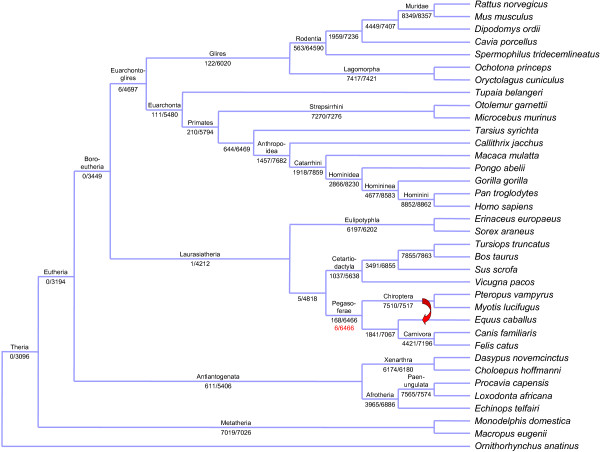
**Mammal phylogeny support through the number of matching gene trees.** For each node, the number of families matching the pattern over the number of eligible families is given. Red arrow shows the branch displacement required to obtain an alternative topology in which *Chiroptera* is a sister group of *E. caballus* and their father node is a sister group of *Carnivora*.

Figure 6 shows the tree topology of the Dos Reis *et al.* phylogeny in which the node level of support is given as the ratio of the number of families matching the pattern over the total number of eligible families (*i.e.*, the HOMOLENS families containing the species found in the subtree). As expected, both numbers decrease as we move from the leaves to the root of the tree due to the fact that deep nodes correspond to subtrees containing a growing number of taxa. A consequence is that the level of support also decreases when moving to the deepest parts of the tree. This goes to an extreme with the nodes corresponding to *Boreoeutheria*, *Eutheria* and *Theria* as they have a support equal to zero in terms of families matching the pattern. This is due to the fact that it is not possible to specify fuzzy patterns in which some species are optional for the search. This would allow to put constraint on the placement of a species in a tree, without rejecting patterns which do not include this species. As we are restricted to strict patterns, finding families containing a large number of species and exactly matching this pattern is difficult.

This method can also be used to test alternative hypotheses in a tree. For instances, the *Pegasoferae* node has the lowest support (168/6466) when looking at all the other nodes located at the same level in the tree. Moreover, Waddell *et al.*[22] made the hypothesis that *Chiroptera* is in fact a sister group of *Equus caballus*. To test this hypothesis we searched for the number of gene families supporting it. For that purpose, we used the pattern ((!//Equuscaballus,(!//Pteropusvampyrus,!//Myotislucifugus)!)!,((!//Canislupusfamiliaris,!//Ailuropodamelanoleuca)!,!//Felis catus)!). With this pattern, we obtained a support level of 6/6466 for *Pegasoferae*, which is significantly lower than the one observed with the original topology.

## Discussion and conclusion

TPMS is a useful set of programs that can be used in a wide range of phylogenetic problems. It has high performance due to its implementation in a compiled language and the fact that all individual programs are multithreaded. Multithreading allows an optimal use on the multi-core architectures that are now common even for desktop computers. For instance, the two pattern searches (with no threshold for bootstrap values) performed on a collection containing 14190 trees to identify the ones supporting the Coelomata or the Ecdyzosoa hypotheses took only six seconds on an Intel Xeon 2.67 GHz with four cores.

For the tree pattern search, the most recent program available to perform this kind of task is PhyloPattern [23]. It is implemented in Java and Prolog and its users have to write their queries as Prolog predicates, which can be very difficult for non-experts. Also, this program cannot be used to root trees or to search for HGTs. Moreover, its performance will be far below the ones of TPMS, due to the fact it is written with interpreted languages.

For the automated tree rooting procedure, different alternatives to the two strategies proposed by TPMS are available. Due to its simplicity, the classical midpoint method looks like a good choice, but this is usually a poor solution. Indeed, midpoint rooting implies that the molecular clock hypothesis is approximately true for the sequences used and, most of the time, this is not the case [24]. Other common approaches are mostly based on algorithms identifying roots that minimize the number of gene duplication and losses, but they require reconciled trees and therefore they need a reliable reference species tree [1,25,26]. In the case of TPMS, the rooting strategy based on the use of an unicity score does not require the availability of such reference tree. This alternative is useful in the case of gene trees containing prokaryotic sequences, as no trusted species tree exist for these organisms. Also, reconciliation methods usually performs poorly with prokaryotic sequences as they do not introduce the possibility of HGTs events.

The application of TPMS for efficient large-scale identification of putative HGTs would require some improvements. Presently, the algorithm is already able to search for incongruent patterns in which a subtree is responsible for a topological shift from the reference species tree. The problem is that the automated procedure provided does not guarantee that the rooting is optimal for prokaryotic species when performing “generic” searches (*e.g.*, searches among the whole Bacteria or Archaea). If there is a lot of HGTs in a tree, our algorithm can lead to a wrong rooting, causing partially wrong taxonomic assignments, and therefore false positives. On the other hand, using TPMS with an explicit transfer pattern (a taxon nested in another when it is not supposed to be according to the species tree) can be very efficient. A method like Prunier, which is specifically devoted to the detection of HGTs is of course more efficient than TPMS in that area but the drawback is that it is limited to unicopy gene families [5].

In the future, we have planned to add functionalities mainly to increase the user-friendliness. For instance, a module automatically completing the species names when building the file containing the associations between sequence names and the species to which they belong is under development. This module works with the remote-acnuc library [27] and requires that the considered species are referenced in the general sequence collections.

## Availability and requirements

**Project name:** TPMS**Project home page:**http://pbil.univ-lyon1.fr/software/tpms/**Operating system(s):** Unix-like operating systems such as Linux and MacOSX (Intel)**Progamming language:** C++**Other requirements:** Bio++ and Boost libraries**License:** GNU GPL

## Competing interests

The authors declare that they have no competing interests.

## Authors’ contributions

TB did all software development, documentation writing and web site set-up, FL participated to conception of the rooting algorithms, VD participated to the incongruencies detection research, GP supervised TPMS development and wrote the manuscript. All authors read and approved the final manuscript.

## Supplementary Material

Additional file 1 **Set of TPMS patterns corresponding to all possible subtrees from the tree shown in Figure **6. Click here for file
